# Supplementary figures and images for: Experimental assessment of diffusible iodine-based contrast-enhanced computed tomography (diceCT) protocols
Source: PeerJ. 2024 Sep 5;12:e17919. doi: 10.7717/peerj.17919 (PMC11380835; doi:10.7717/peerj.17919)

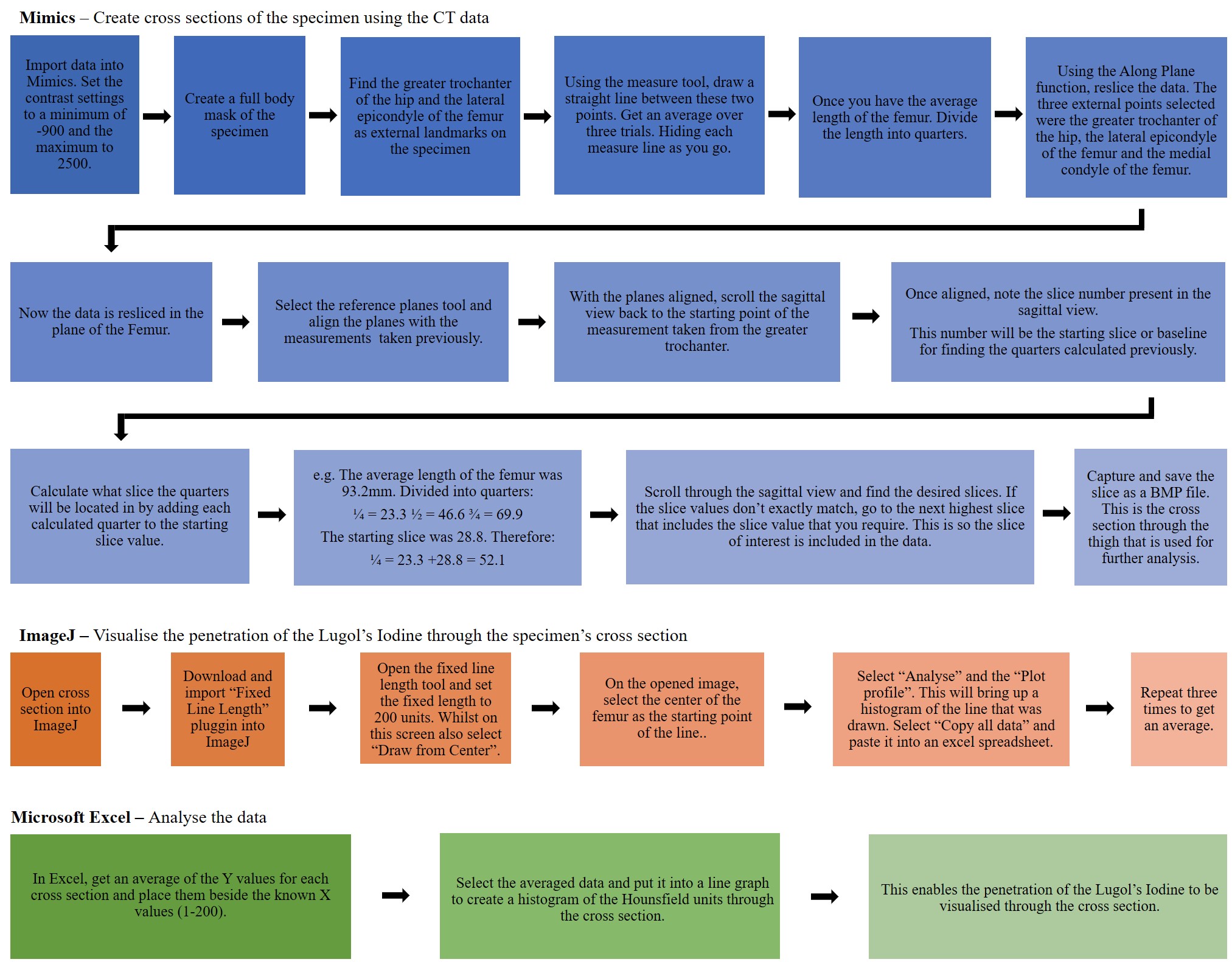

Supplement: Supplemental Information 1 — The steps of quantifying the data obtained from CT scans using three programs; Materialise Mimics, ImageJ and Microsoft Excel. [file peerj-12-17919-s001.jpg]

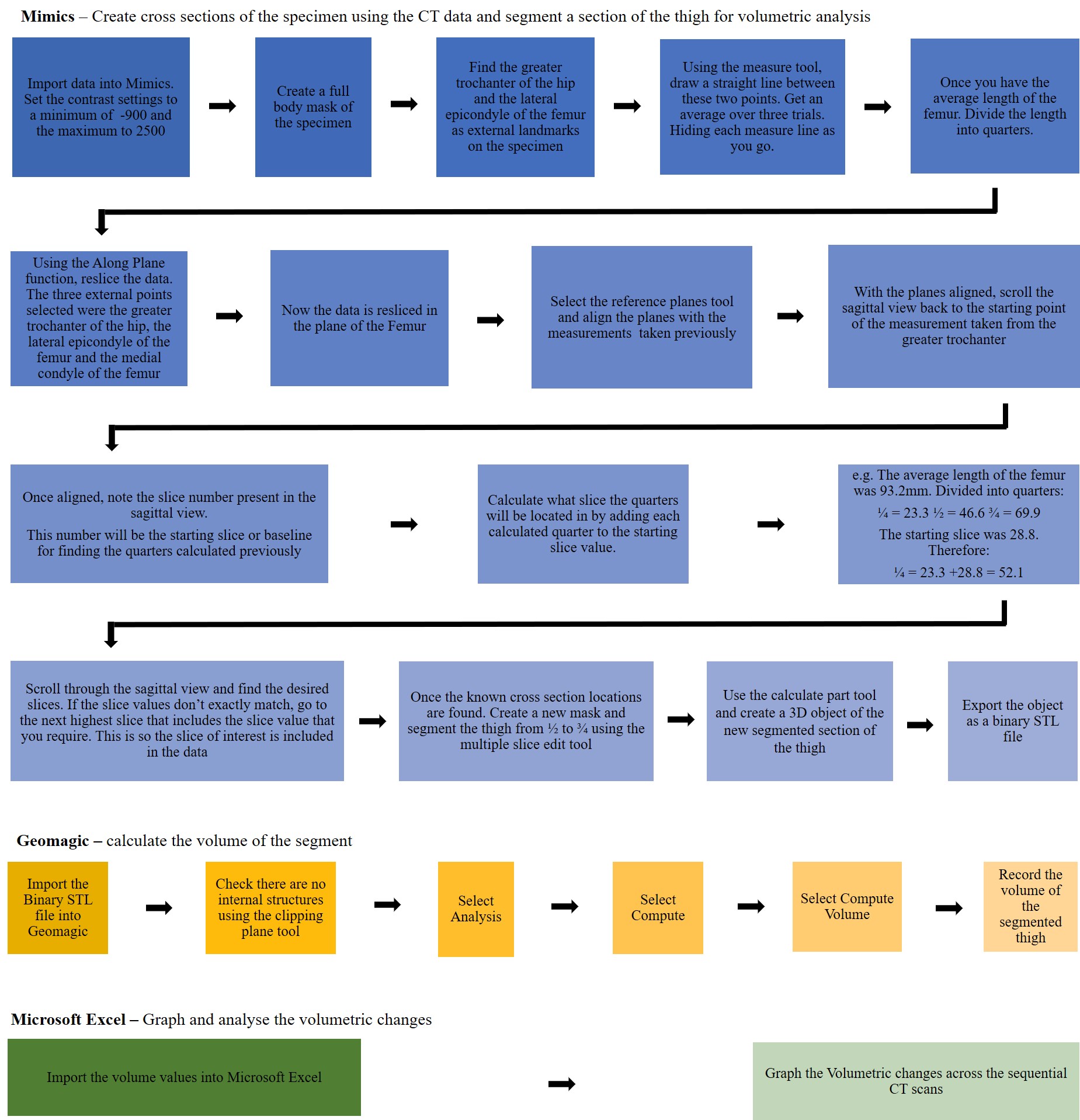

Supplement: Supplemental Information 2 — The steps of quantifying shrinkage from the data obtained from the CT scans using three programs; Materialise Mimics, Geomagic and Microsoft Excel [file peerj-12-17919-s002.jpg]

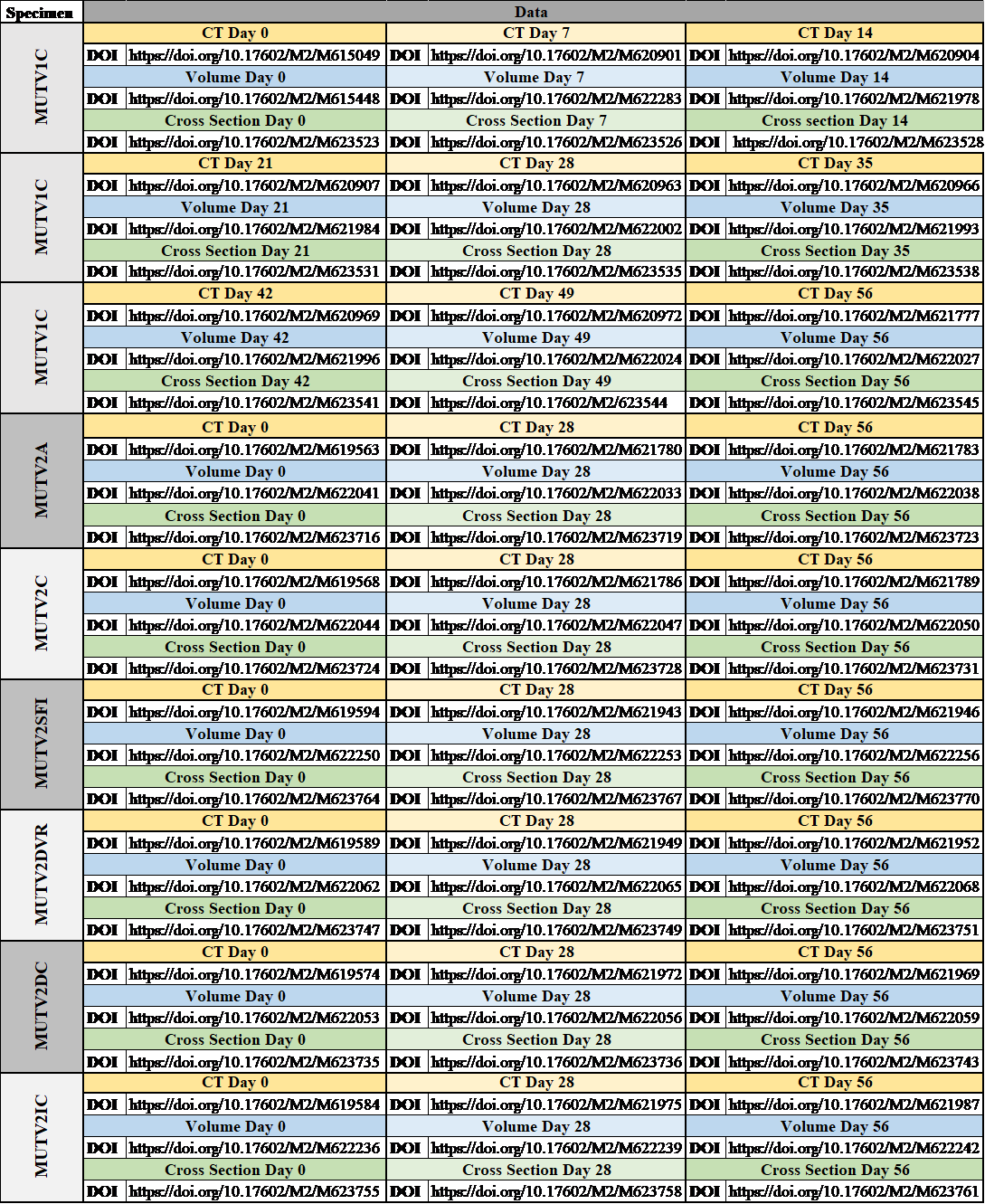

Supplement: Supplemental Information 3 — Morphosource project “Experimental Assessment of Diffusible Iodine-Based Contrast-Enhanced Computed Tomography (DiceCT) Protocols Datasets” DOIs for all CT, volumetric and cross section data across the seven specimens MUTV1C, MUTV2A, MUTV2C, MUTV2SFI, MUTV2DVR, MUTV2DC and MUTV2IC. [file peerj-12-17919-s003.png]
